# RETRACTED: Chen et al. LncRNA ERVH48-1 Contributes to the Drug Resistance of Prostate Cancer and Proliferation through Sponging of miR-4784 to the Activation of the Wnt/β-Catenin Pathway. *Cancers* 2023, *15*, 1902

**DOI:** 10.3390/cancers18081229

**Published:** 2026-04-13

**Authors:** Binshen Chen, Kai Xu, Yiming Zhang, Peng Xu, Chaoming Li, Jun Liu, Yawen Xu

**Affiliations:** 1 Department of Urology, Zhujiang Hospital, Southern Medical University, Guangzhou 510282, China; 2 Guangzhou Key Laboratory of Inflammatory and Immune Diseases, Zhujiang Hospital, Southern Medical University, Guangzhou 510282, China

The journal retracts the article titled “LncRNA ERVH48-1 Contributes to the Drug Resistance of Prostate Cancer and Proliferation Through Sponging of miR-4784 to the Activation of the Wnt/β-Catenin Pathway” [1], cited above.

Following publication, concerns were brought to the attention of the Editorial Office regarding the integrity of a figure published in this article [1].

In accordance with standard journal procedures, the Editorial Office and Editorial Board conducted an investigation that confirmed an overlap between Figure 7G in [1] and Figure 2F in a previously published article [2], produced by a different research group and presenting different experimental conditions. Although the authors cooperated with the investigation, the explanations and supporting materials provided were not deemed sufficient by the Editorial Board to resolve the identified concerns. As a result, the Editorial Board has lost confidence in the reliability of the findings, and has decided to retract this publication, as per MDPI’s retraction policy.

This retraction was approved by the Editor-in-Chief of the journal *Cancers*.

The authors have been informed of this retraction but did not provide a comment on this decision.
